# Evaluating Specimen Quality and Results from a Community-Wide, Home-Based Respiratory Surveillance Study

**DOI:** 10.1128/JCM.02934-20

**Published:** 2021-04-20

**Authors:** Ashley E. Kim, Elisabeth Brandstetter, Naomi Wilcox, Jessica Heimonen, Chelsey Graham, Peter D. Han, Lea M. Starita, Denise J. McCulloch, Amanda M. Casto, Deborah A. Nickerson, Margaret M. Van de Loo, Jennifer Mooney, Misja Ilcisin, Kairsten A. Fay, Jover Lee, Thomas R. Sibley, Victoria Lyon, Rachel E. Geyer, Matthew Thompson, Barry R. Lutz, Mark J. Rieder, Trevor Bedford, Michael Boeckh, Janet A. Englund, Helen Y. Chu

**Affiliations:** a Department of Medicine, University of Washington, Seattle, Washington, USA; b Brotman Baty Institute for Precision Medicine, Seattle, Washington, USA; c Department of Genome Sciences, University of Washington, Seattle, Washington, USA; d Formative, Seattle, Washington, USA; e Vaccine and Infectious Disease Division, Fred Hutchinson Cancer Research Center, Seattle, Washington, USA; f Seattle Children’s Research Institute, Seattle, Washington, USA; g Department of Family Medicine, University of Washington, Seattle, Washington, USA; h Department of Bioengineering, University of Washington, Seattle, Washington, USA; University of Iowa College of Medicine

**Keywords:** influenza, respiratory pathogens, rapid diagnosis, nasal swab, pandemic preparedness

## Abstract

While influenza and other respiratory pathogens cause significant morbidity and mortality, the community-based burden of these infections remains incompletely understood. The development of novel methods to detect respiratory infections is essential for mitigating epidemics and developing pandemic-preparedness infrastructure. From October 2019 to March 2020, we conducted a home-based cross-sectional study in the greater Seattle, WA, area, utilizing electronic consent and data collection instruments. Participants received nasal swab collection kits via rapid delivery within 24 hours of self-reporting respiratory symptoms. Samples were returned to the laboratory and were screened for 26 respiratory pathogens and a housekeeping gene. Participant data were recorded via online survey at the time of sample collection and 1 week later. Of the 4,572 consented participants, 4,359 (95.3%) received a home swab kit and 3,648 (83.7%) returned a nasal specimen for respiratory pathogen screening. The 3,638 testable samples had a mean RNase P relative cycle threshold (*C_rt_*) value of 19.0 (SD, 3.4), and 1,232 (33.9%) samples had positive results for one or more pathogens, including 645 (17.7%) influenza-positive specimens. Among the testable samples, the median time between shipment of the home swab kit and completion of laboratory testing was 8.0 days (interquartile range [IQR], 7.0 to 14.0). A single adverse event occurred and did not cause long-term effects or require medical attention. Home-based surveillance using online participant enrollment and specimen self-collection is a safe and feasible method for community-level monitoring of influenza and other respiratory pathogens, which can readily be adapted for use during pandemics.

## Abstract (precis)

While influenza and other respiratory pathogens cause significant morbidity and mortality, the community-based burden of these infections remains incompletely understood. The development of novel methods to detect respiratory infections is essential for mitigating epidemics and developing pandemic-preparedness infrastructure.

## INTRODUCTION

Acute respiratory illnesses (ARIs) constitute a significant burden on the health care system in the United States and represent an important cause of morbidity and mortality worldwide (1–4). In the United States, influenza causes 140,000 to 810,000 hospitalizations and 12,000 to 67,000 deaths annually (1–4). Additionally, respiratory syncytial virus (RSV) leads to approximately 2 million outpatient visits each year for children under the age of 5 (https://www.cdc.gov/flu/about/burden/index.html) (5). Estimates of the prevalence of ARI-causing pathogens generally rely on in-person health care visits or aggregate counts from hospitalized individuals (https://www.cdc.gov/flu/weekly/overview.htm) (5–8). Thus, these estimates likely omit cases of mild to moderate ARI in community-dwelling individuals who may not seek care for their illness (9–11).

Active, community-level monitoring of respiratory infections is essential to assess the seasonal activity of ARI-causing pathogens and can be used to inform public health prevention strategies and influence treatment decisions made at the community level. Previous respiratory pathogen surveillance studies evaluated specific subsets of the population, such as households with children, or used labor-intensive, coordinated efforts to capture a representative sample of the community, which makes such approaches difficult to replicate (12–14). Additionally, similar to traditional respiratory surveillance networks, some of these studies relied on health care facility visits which have the potential to result in the nosocomial spread of respiratory pathogens (15, 16). Despite the limitations of earlier analyses, community-wide surveillance studies remain of vital importance, as they provide opportunities to better understand the epidemiology of respiratory illness among symptomatic individuals with variable disease severities and health care-seeking behaviors.

The Seattle Flu Study (SFS) “Swab and Send” is a novel, city-wide, cross-sectional study of home-based detection of respiratory pathogens. This study demonstrates the feasibility of using a home-based surveillance approach to assess the epidemiology of influenza and other respiratory pathogens in a community-based setting.

## MATERIALS AND METHODS

### Study design.

The Swab and Send study was nested within the Seattle Flu Study (SFS), a multiarmed influenza surveillance system (17). This study aimed to assess the feasibility of city-wide home-based cross-sectional respiratory pathogen surveillance, utilizing rapid delivery systems for at-home collection of a nasal swab from individuals experiencing ARIs with return of specimens to the laboratory for respiratory pathogen detection. Individuals residing within the greater Seattle, WA, area with ARI symptoms were prospectively enrolled from October 2019 to March 2020. Participants resided in 89 different zip codes within King County in and around Seattle, WA. This study was approved by the University of Washington Institutional Review Board.

### Recruitment.

Study recruitment occurred through (i) referrals from health care providers, clinics, Seattle Flu Study community kiosks (an in-person enrollment center), schools, and workplaces; (ii) dissemination of printed flyers posted at community locations; and (iii) posting of targeted online advertisements (e.g., Facebook, Instagram, Twitter, and Google). Recruitment materials directed potential participants to the study website (www.seattleflu.org, henceforth referenced as the “study website”). To determine their eligibility, individuals completed a screening survey on the study website by providing their age, home zip code, and information about the presence and duration of respiratory symptoms and by verifying their access to the Internet.

Individuals were eligible to participate in the study if they lived within the specified zip codes, had experienced new or worsening cough and/or two ARI symptoms (subjective fever, headache, sore throat or itchy/scratchy throat, nausea or vomiting, runny/stuffy nose or sneezing, fatigue, muscle or body aches, increased trouble with breathing, diarrhea, ear pain/discharge, or rash) within 7 days of enrollment (see Table SA1 in the supplemental material), were English speaking, had a valid email address, and had access to the Internet at home. All individuals consented to participate in the research study electronically, with consent by a parent or legally authorized representative for individuals under 18 years and concurrent assent for those between 7 and 18 years.

### Data collection.

Upon consenting, participants completed an online enrollment questionnaire to provide their home address and contact information, such as an email address or phone number. Participants were mailed a home swab kit within 48 hours of submitting the enrollment questionnaire, which included a Quick Start Instruction Card (see Fig. SA1 in the supplemental material), a universal viral transport medium (UTM) tube (Becton, Dickinson and Company, Sparks, MD), a nylon flocked midturbinate swab (Copan Diagnostics Inc., Murietta, CA), a return box with an affixed category B UN3373 label (as required by International Air Transport Association [IATA] guidelines; https://www.un3373.com/category-biological-substances/category-b/), and a prepaid return shipping label. Pediatric nasal swabs (Copan Diagnostics Inc.) were available for participants 5 years of age or younger. Various couriers were used to deliver home swab kits to participants across King County, depending on geographical location as determined by zip code. For the 2,398 of participants who resided within Seattle, WA, FedEx Same Day City was used to deliver kits with a target delivery time of 2 hours.

Upon kit receipt, participants completed an online illness questionnaire to ascertain demographics, illness characteristics, and health behaviors. Education level was only asked of participants 18 and older. Additionally, participants were asked to rate the impact of their current illness on regular activities at the time of their enrollment using a five-point Likert scale with the following levels: not at all, a little bit, somewhat, quite a bit, or very much. These categories were transformed into none, low (a little bit, somewhat), and high (quite a bit, very much).

At the end of the illness questionnaire, participants were prompted to self-collect a midnasal swab using instructions on the Quick Start Instruction Card (see Fig. S1 in the supplemental material) included in the swab kit box. Participants were instructed to place their self-collected nasal swabs directly into the UTM tube which was prelabeled with a unique sample barcode. Next, participants were instructed to place the UTM tube containing the self-collected nasal swab into a specimen bag, prepackaged with an absorbent sheet, and then to put the specimen bag into the provided return shipping box. United States Postal Service (USPS) return postage and category B UN3373 stickers were affixed to outside the return box. Although previous testing has demonstrated that respiratory viral RNA is stable at room temperature in UTM for up to 1 week (18), participants were encouraged to return their nasal specimen within 24 hours or as soon as possible. For the subset of participants where detailed courier data were available, median delivery times were determined through the use of proof-of-delivery (POD) data on scheduled shipment times, completed delivery times, and mileage.

Seven days after nasal swab collection, participants were recontacted to complete a 1 week follow-up questionnaire to assess the impact of their illness on health care-seeking behaviors. Care seeking was marked as “any care” if the participant indicated they had sought care in the illness questionnaire or 1 week follow-up questionnair*e*. Any care seeking included doctor’s office or urgent care, pharmacy, hospital or emergency department, or other.

### Laboratory testing.

When kits arrived in the study laboratory, the contents of the box and deviations from return mail instructions were recorded. A total of 200 μl of UTM was removed and subjected to RNA extraction using a MagNA Pure 96 system (Roche), and the remainder was banked at −80°C. The extracted nucleic acids were screened for respiratory pathogens using a custom, TaqMan-based Open Array panel (Thermo Fisher) and an additional severe acute respiratory syndrome coronavirus 2 (SARS-CoV-2) reverse transcriptase PCR (RT-PCR) research assay (https://assets.thermofisher.com/TFS-Assets/LSG/manuals/MAN0017952_RespiratoryTractMicrobiotaProfiling_OA_AG.pdf). Samples were subjected to the SARS-CoV-2 assay in real time if they were collected after 25 February 2020 and retrospectively if collected between 1 January 2020 and 24 February 2020 (see Table SA2 in the supplemental material) (19). Samples with RNase P relative cycle threshold (*C_rt_*) values of ≤28 for the Open Array assay, as recommended by Thermo Fisher, which has a preamplification step, and ≤36 for the SARS-CoV-2 assay were considered to contain sufficient material for pathogen detection (https://assets.thermofisher.com/TFS-Assets/LSG/manuals/MAN0017952_RespiratoryTractMicrobiotaProfiling_OA_AG.pdf). The RNase P *C_r_t* cutoff for the SARS-CoV-2 laboratory-developed test was determined by repeat testing of contrived positive samples near the limit of detection. Unlike the threshold cycle (*C_T_*) method which considers all the amplification curves for a specific target to determine the threshold, the *C_rt_* method sets a threshold for each curve individually that is determined by the shape of the amplification curve regardless of the height or variability of the curve in its early baseline fluorescence. Samples were screened for influenza A H3N2 and H1N1; pan influenza A, influenza B, and influenza C; respiratory syncytial viruses (RSVs) A and B; human coronaviruses (hCoVs) 229E, NL63, OC43, and HKU1; SARS-CoV-2; adenovirus (AdV); human rhinovirus (hRV); human metapneumovirus (hMPV); human parechovirus (hPeV); enteroviruses A, B, C, D, D68, and G; human bocavirus (hBoV); Streptococcus pneumoniae; Mycoplasma pneumoniae; and Chlamydia pneumoniae (Table SA2). *C_r_t* values for RNase P, influenza, hCoV, RSV, and hRV from 11,984 nasal samples collected between October 2019 and March 2020 at Seattle Children’s Hospital were analyzed as a contemporary control of health care worker-collected specimens and compared with the self-collected specimens in this study.

**TABLE 2:** Clinical and sociodemographic characteristics of enrolled participants from 16 October 2019 to 9 March 2020 by study procedure completion and compliance^*a*^

Characteristic	Study procedure completion		Study procedure compliance				
	Returned nasal swab (*n* = 3,638)	Completed all study procedures (*n* = 3,214)	Mail packaging error^*b*^ (*n* = 205)	Sample tube use error^*c*^ (*n* = 24)	Sample tube labeling error^*d*^ (*n* = 205)	No packaging or sample tube errors (*n* = 3,211)	*P* value^*e*^
Age (yrs)	0.11
<5	110 (3.0)	89 (2.8)	9 (4.4)	1 (4.2)	6 (2.9)	92 (2.9)
5–17	173 (4.8)	149 (4.6)	12 (5.9)	0 (0)	16 (7.8)	149 (4.6)
18–49	2,638 (72.5)	2,324 (72.3)	144 (70.2)	15 (62.5)	141 (68.8)	2,339 (72.8)
50–64	545 (15.0)	496 (15.4)	33 (16.1)	6 (25.0)	29 (14.1)	480 (14.9)
≥65	168 (4.6)	153 (4.8)	6 (2.9)	2 (8.3)	10 (4.9)	150 (4.7)
Sex	0.38
Male	1,142 (31.4)	1,013 (31.5)	70 (34.1)	8 (33.3)	70 (34.1)	994 (31.0)
Female	2,340 (64.3)	2,178 (67.8)	115 (56.1)	13 (54.2)	118 (57.6)	2,097 (65.3)
Other	18 (0.5)	15 (0.5)	3 (1.5)	0 (0)	1 (0.5)	14 (0.4)
Income	0.81
≤$25,000	180 (4.9)	161 (5.0)	5 (2.5)	1 (4.2)	9 (4.4)	164 (5.1)
$25,000–50,000	344 (9.5)	315 (9.8)	21 (10.3)	2 (8.3)	27 (13.2)	294 (9.2)
$50,000–100,000	818 (22.5)	760 (23.6)	39 (19.0)	10 (41.7)	49 (23.9)	716 (22.3)
$100,000–150,000	700 (19.2)	639 (19.9)	33 (16.1)	2 (8.3)	32 (15.6)	635 (19.8)
≥$150,000	1,129 (31.0)	1,042 (32.4)	69 (33.7)	6 (25.0)	48 (23.4)	1,010 (31.5)
Education level	0.53
Graduated high school/obtained GED or less	101 (2.8)	80 (2.5)	9 (4.4)	0 (0)	10 (4.9)	81 (2.5)
Some college (including vocational training, associate’s degree)	449 (12.3)	414 (12.9)	20 (9.8)	5 (20.8)	32 (15.6)	395 (12.3)
Bachelor’s degree	1,324 (36.4)	1,220 (38.0)	67 (32.7)	5 (20.8)	58 (28.3)	1,189 (37.0)
Advanced degree	1,328 (36.5)	1,229 (38.2)	68 (33.2)	10 (41.7)	66 (32.2)	1,188 (37.0)
Care-seeking	0.80
Any care prior to enrollment or during study period	1,138 (31.3)	1,077 (33.5)	52 (25.4)	7 (29.2)	63 (30.7)	1,013 (31.5)
No care prior to enrollment or during study period	2,136 (58.7)	2,136 (66.5)	114 (55.6)	13 (54.2)	105 (51.2)	1,912 (59.5)
Illness impact on regular activities at enrollment	0.07
None	234 (6.4)	203 (6.3)	17 (8.3)	1 (4.2)	11 (5.4)	205 (6.4)
Low	1,521 (41.8)	1,373 (42.7)	90 (43.9)	10 (41.7)	73 (35.6)	1,345 (41.9)
High	1,754 (48.2)	1,637 (50.9)	81 (39.5)	10 (41.7)	107 (52.2)	1,564 (48.7)

a All values are no. of participants (%).

b Mail packaging errors include returning the nasal specimen in a damaged box, a different box than the one provided, an improperly closed box, or an improperly used specimen transport bag or lack thereof.

c Sample tube use errors include returned nasal specimens with a damaged or broken UTM tube, an absent swab, or leakage.

d Sample tube labeling errors include a missing written full name or date of collection on the UTM tube.

e Kruskal-Wallis test was used to determine *P* values for study procedure compliance categories (excludes first three columns).

### Data analyses.

Descriptive statistics were performed for categorical and continuous covariates. Bivariate analyses were conducted using parametric and nonparametric tests as appropriate, with statistical significance defined as a *P* value of <0.05. The Kruskal-Wallis test was used to determine *P* values for study procedure compliance categories, comparing each of the three nasal swab error types to those with no errors. Analysis of variance (ANOVA) was used to calculate an overall *P* value for RNase *P* values across confidence and discomfort levels. Respiratory pathogen prevalence is defined as the total number of cases detected out of the total number of tested samples.

## RESULTS

### Participant characteristics.

A total of 4,572 participants consented and were enrolled in the SFS Swab and Send study from 16 October 2019 to 9 March 2020. The majority of participants were recruited into the study through online or social media advertisements (53.9%) or through referrals from friends or family (19.3%). Of the 4,572 participants who completed the electronic consent form, 4,359 (95.3%) participants also completed the enrollment questionnaire and provided a valid home address, which was required to receive a home swab kit. Participant characteristics, including age, sex, race, Hispanic ethnicity, income, education level, influenza vaccination status, health care-seeking status, test results, baseline impact of illness on regular activities, and recruitment method are shown in Table 1. The mean age of study participants was 36.6 (SD, 15.0) years old. Most participants (73.7%) were 18 to 49 years old. On average, the study population was more highly educated and had a higher household income than the general population of King County. A total of 31.4% of participants had a bachelor’s degree as their highest degree, while 31.6% had an advanced degree. A total of 26.6% had a household income of ≥$150,000 per year (Table 1).

**TABLE 1:** Clinical and sociodemographic characteristics of enrolled participants from 16 October 2019 to 9 March 2020

Characteristic	No. (%) of participants^*a*^
Age (yrs)
<5	128 (2.9)
5–17	208 (4.8)
18–49	3,212 (73.7)
50–64	614 (14.1)
≥65	192 (4.4)
Sex
Male	1,191 (27.3)
Female	2,451 (56.2)
Other	19 (0.4)
Race
American Indian/Alaska Native	17 (0.4)
Asian	724 (16.6)
Native Hawaiian/Pacific Islander	7 (0.2)
Black/African American	37 (0.8)
White	2,542 (58.3)
Other	92 (2.1)
Multiple	188 (4.3)
Hispanic ethnicity (*n* = 2,856)	183 (4.2)
Income
≤$25,000	196 (4.5)
$25,000–50,000	367 (8.4)
$50,000–100,000	860 (19.7)
$100,000–150,000	738 (16.9)
≥$150,000	1,160 (26.6)
Education level
Graduated high school/obtained GED or less	109 (2.5)
Some college (including vocational training, associate’s degree)	492 (11.3)
Bachelor’s degree	1,371 (31.5)
Advanced degree	1,377 (31.6)
Care-seeking
Any care prior to enrollment or during study period	1,182 (27.1)
No care prior to enrollment or during study period	2,183 (50.1)
Illness impact on regular activities at enrollment
None	243 (5.6)
Low	1,597 (36.6)
High	1,831 (42.0)
How participant heard about the study
Saw an ad on Facebook/Instagram/Twitter	1,369 (31.4)
Referral from a friend/family member	841 (19.3)
Other online	667 (15.3)
Saw an ad on Google	314 (7.2)
Referral from my place of work	280 (6.4)
Other	172 (3.9)
Saw a Seattle Flu Study kiosk	86 (2.0)
Email/Seattle Community Pulse	86 (2.0)
Referral from a healthcare provider, travel clinic, or immigrant/refugee health screening	60 (1.4)
Referral from my child’s school	29 (0.7)

a Total *n* = 4,359.

At the time of enrollment, 42.0% of participants who were sent a nasal swab rated the impact of their current illness on their regular activities as high, although 67.5% had not sought clinical care. The majority of study participants did not seek clinical care for their illness during the study period. A total of 27.1% of participants sought clinical care for their current illness prior to enrollment or during the study period, whereas 50.1% never sought clinical care during this time frame (Table 1). In general, participants who sought care were more likely to do so after enrolling and completing their home swab kits. Among those who sought care (*n* = 1,178), 727 (61.7%) participants sought care prior to enrollment and 989 (84.0%) sought care within 1 week after enrollment, although these categories are not mutually exclusive.

**FIG 1:**
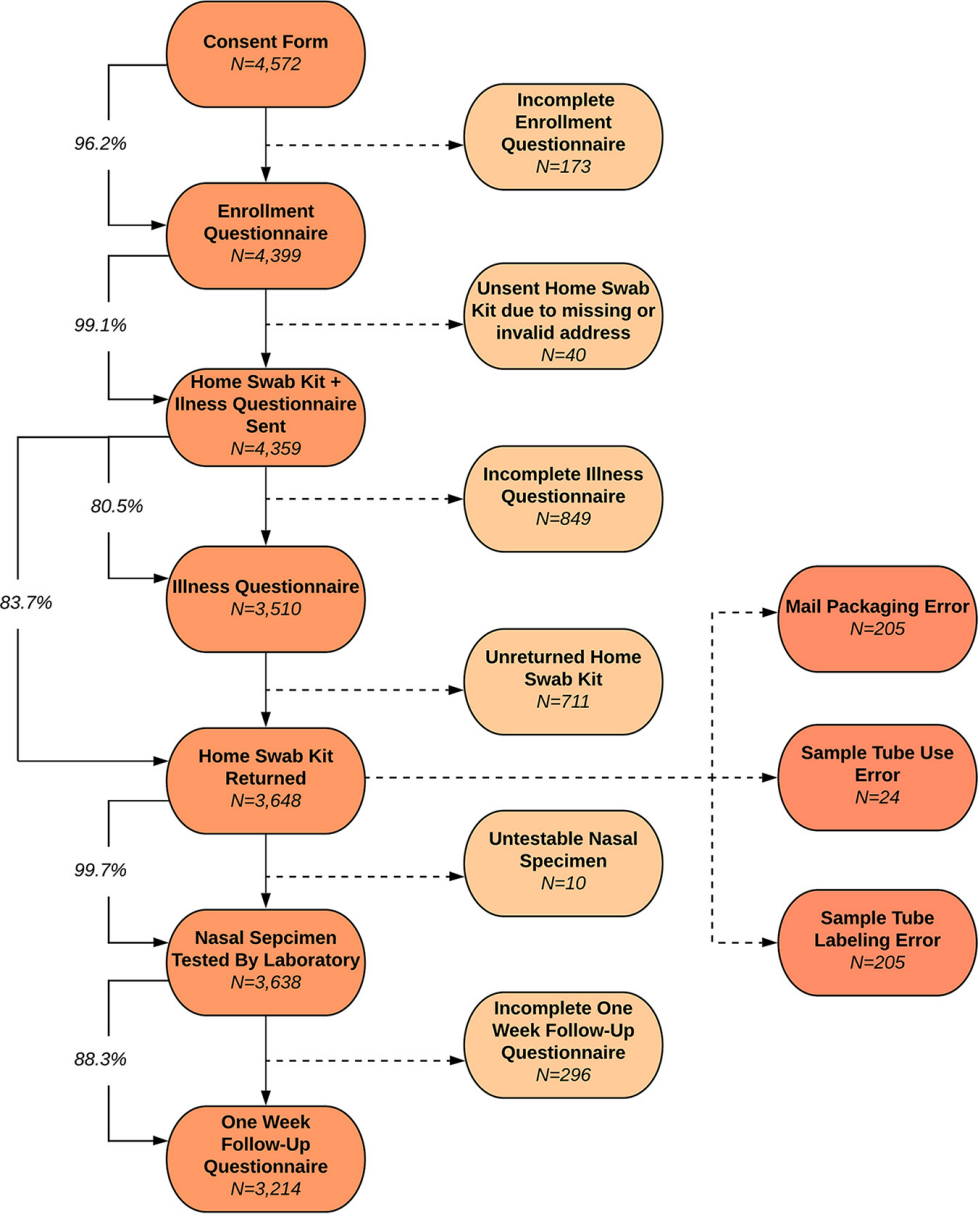
Study procedure completion rates. Mail packaging errors included a damaged box, a different box used than the one provided, an improperly closed box, or an improperly used specimen transport bag or lack thereof. Sample tube use errors included damaged or broken UTM tube, an absent swab, or leakage. Sample tube labeling errors included a missing written full name or date of collection on the UTM tube.

**FIG 2:**
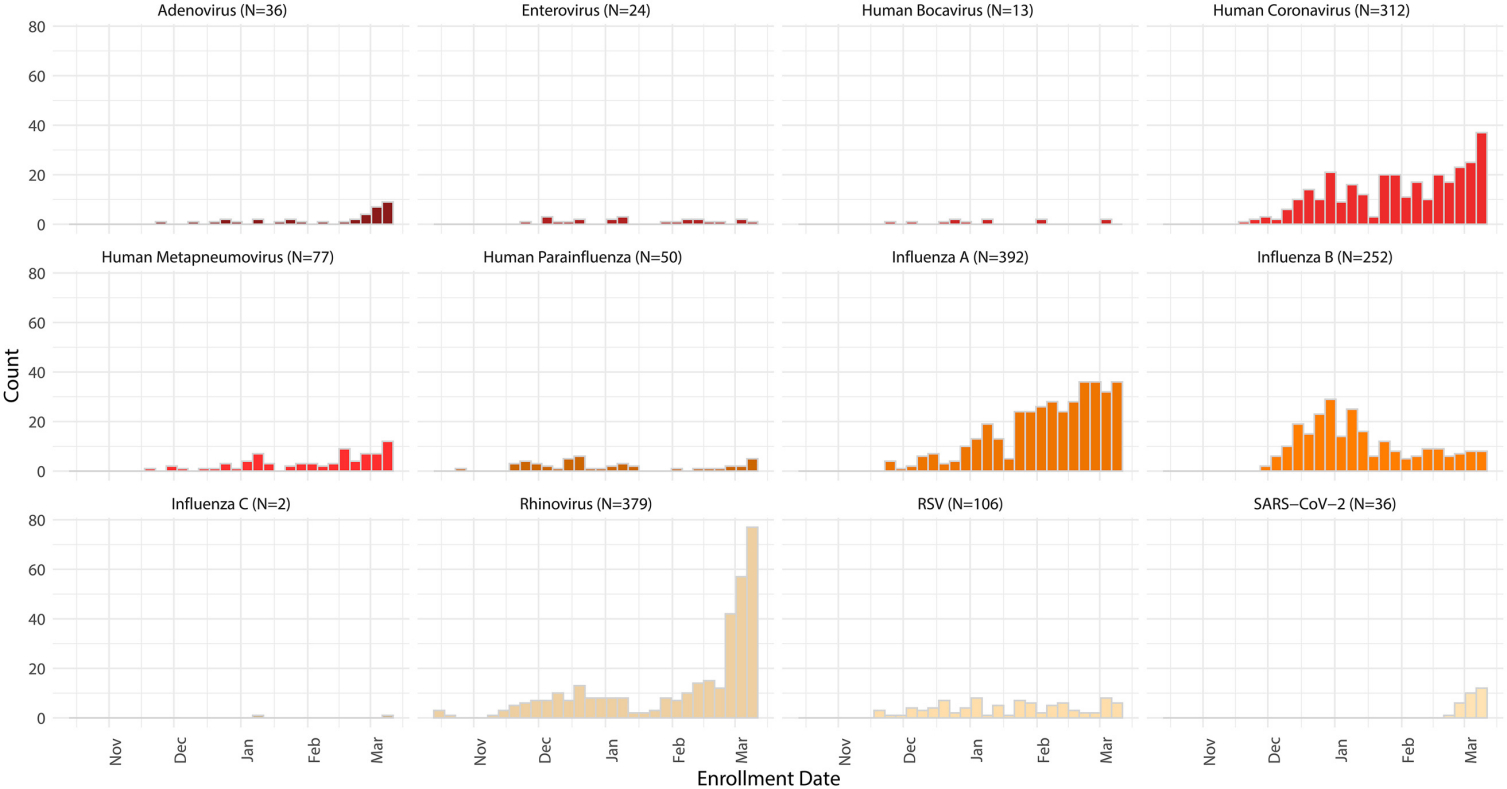
Pathogens detected in participants over time from 16 October 2019 to 9 March 2020.

Of the 4,359 participants who received a home swab kit, 3,648 (83.7%) returned a nasal specimen to the laboratory and 3,638 (99.7%) of returned specimens contained sufficient UTM in the tube and RNase P levels for respiratory pathogen screening (Fig. 1). Influenza A (10.8%), hRV (10.4%), hCoV (8.6%), and influenza B (6.9%) were the most commonly detected pathogens (see Table SA3 in the supplemental material; Fig. 2). Samples collected on or after 1 January 2020 were tested for SARS-CoV-2, of which 36 out of 2,843 (1.2%) were positive for the novel coronavirus. The 3,629 self-collected nasal specimens with available RNase P data yielded a mean RNase P relative cycle threshold (*C_rt_*) value of 19.0 (SD, 3.4) (Table SA3). A contemporary comparison of *C_rt_* values from health care worker-collected nasal specimens to self-collected nasal specimens is shown in Table SA4 in the supplemental material. The average *C_rt_* values of health care worker-collected nasal samples were lower than those of the self-collected nasal samples for RNase P, influenza, and RSV. In contrast, the average *C_rt_* values of self-collected nasal samples were lower than those of the health care worker-collected nasal samples for hCoV and hRV (Table SA4).

### Study logistics.

**FIG 3:**
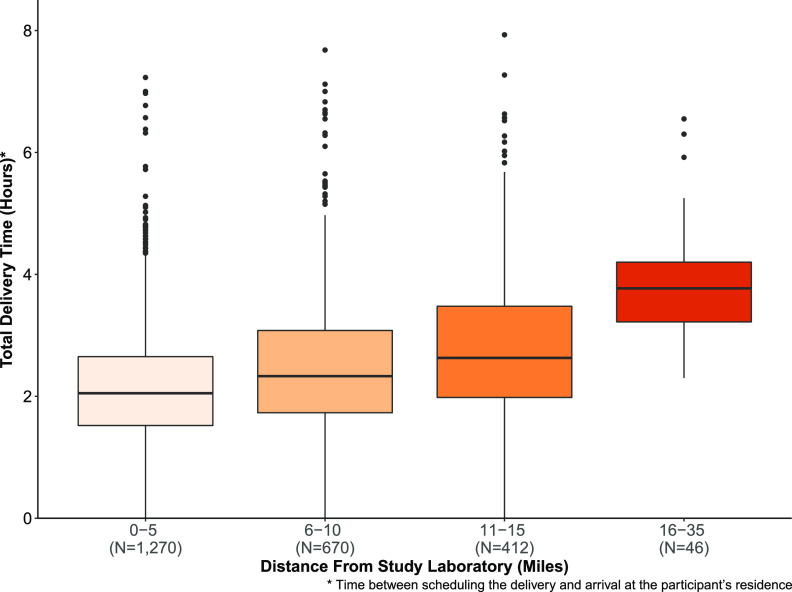
Median delivery times of home swab kits to participants by distance from study laboratory (*n* = 2,398).

For the 4,359 participants who received a home swab kit, the median time between participant completion of enrollment and scheduling of the shipment was 7.2 hours (interquartile range [IQR], 0.45 to 19.6]. The total median delivery transit time to participants who received their home swab kit via FedEx Same Day City was 2.2 (IQR, 1.7 to 3.0) hours, with 79% of deliveries meeting the 2-hour target delivery time. A subset of the delivery time data was reported previously (22). The median delivery time via FedEx Same Day City to participants’ homes by distance from the study laboratory is shown in Fig. 3. Of the 2,398 FedEx Same Day City deliveries, there were a total of 78 (3.3%) redelivery attempts. The estimated median time between nasal swab collection to receipt at the study laboratory was 3.0 (IQR, 2.0, 4.0) days for the 3,648 participants who returned specimens. Of the 3,638 testable samples, the median time between shipment and completed laboratory testing was 8.0 (IQR, 7.0 to 14.0) days.

### Study procedure completion and compliance.

Study procedure completion rates are shown in Fig. 1. Of the 4,359 participants who completed the enrollment questionnaire and received a home swab kit, 3,214 (73.9%) completed all study procedures. Study procedure completion and compliance by age, sex, income, education, care-seeking status, and baseline illness impact are shown in Table 2. None of these variables were significantly associated with study procedure compliance (Table 2).

The majority of participants correctly followed instructions to package their collected nasal swab for return to the laboratory. Of the 3,648 returned nasal specimens, 3,208 (88.1%) home swab kits were returned correctly packaged. A total of 205 (5.6%) contained a sample tube labeling error, such as a missing written name or collection date, and 205 (5.6%) were mispackaged. Criteria for mispackaged samples included improper use of the provided return box, specimen transport bag, or lack thereof. Additionally, 24 (0.66%) returned specimens had a sample tube use error, such as a damaged UTM tube, a missing or misused nasal swab, or leakage. Four out of 3,648 (0.11%) returned home swab kits contained leakage, and these samples were immediately disposed of upon unpackaging (Table 2).

**FIG 4:**
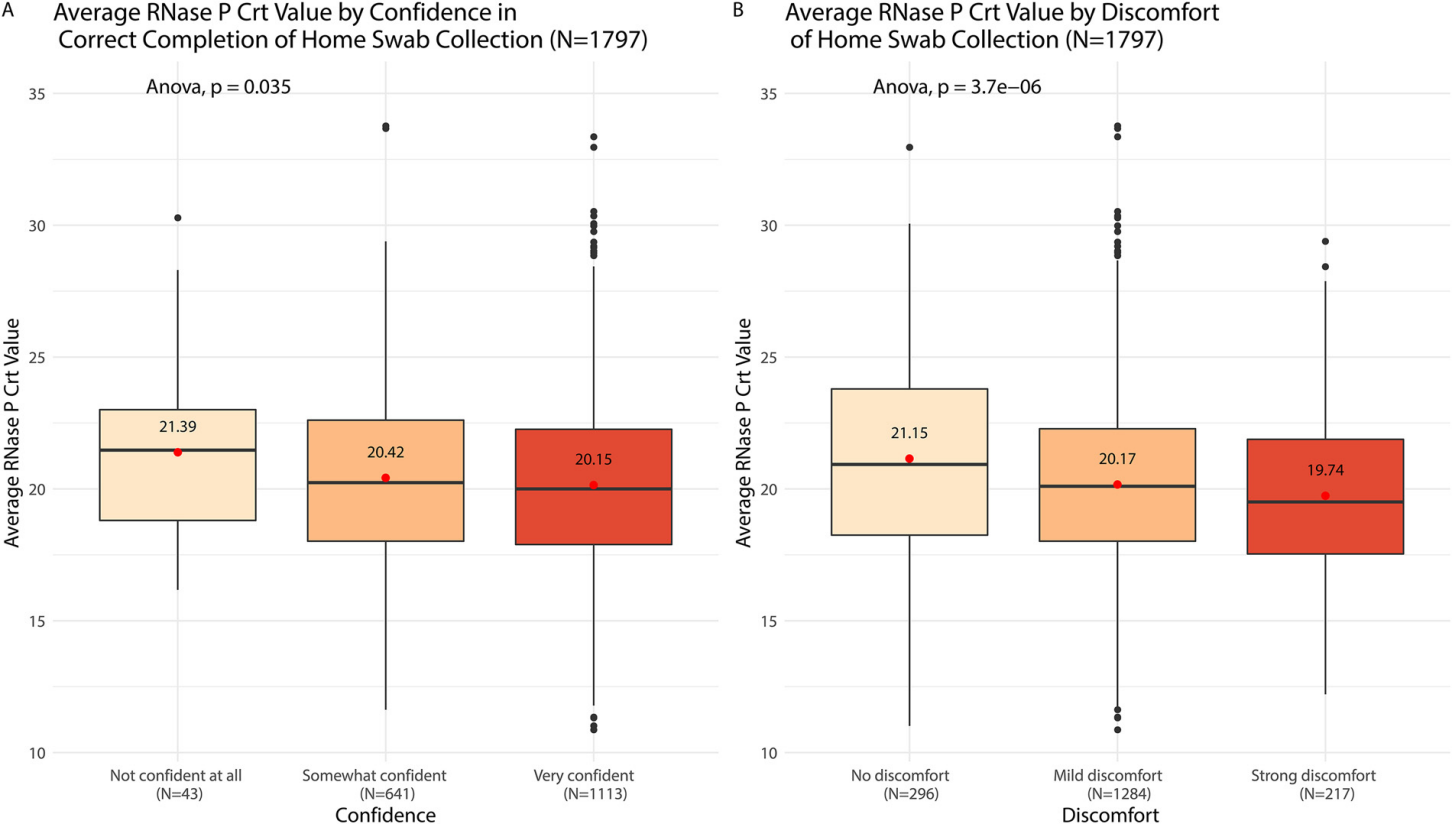
Average RNase P *C_rt_* values by discomfort of and confidence in home swab collection. Participants (*n* = 1,796) who enrolled from 6 January 2020 to 9 March 2020 were asked to rate their confidence in the correct completion of the home swab (not confidence at all, somewhat confident, or very confident) and their discomfort in the collection of the home swab (no discomfort, mild discomfort, or strong discomfort).

Participants who enrolled between 6 January 2020 and 9 March 2020 were asked to rate their confidence in correctly self-collecting their nasal swab and their discomfort level while doing so. Higher confidence and discomfort levels were significantly associated with lower RNase P *C_rt_* values (*P* < 0.001 and *P* = 0.04, respectively). The average RNase P *C_rt_* value for participants who experienced strong discomfort was 1.4 lower than the average value for those who had no discomfort. The average RNase P *C_rt_* value for those who were very confident was 1.2 lower than those who were not confident at all (Fig. 4). Among the 4,359 participants who received a home swab kit, there was one (<0.01%) who reported adverse event related to strong discomfort while collecting the nasal swab. The affected participant’s discomfort resolved within 2 min. The participant suffered no long-term effects and did not require medical attention. Results suggest that nonmedically trained individuals can safely and adequately collect a nasal sample from themselves or their family members.

## DISCUSSION

Over the 2019 to 2020 influenza season, we enrolled a large cohort of participants with acute respiratory illness in a study of home-based swab collection for detection of respiratory pathogens. The majority of participants completed all study procedures and returned their nasal specimens to the study laboratory in a timely manner and in compliance with federal transport guidelines for biohazards. The majority of returned nasal specimens were adequately self-collected as quantified by RNase P *C_rt_* values. These results support the feasibility of using online enrollment and self-collected nasal swabs for community surveillance of respiratory pathogens.

Existing methods to estimate the community-level prevalence of influenza rely on estimator models based on laboratory-confirmed cases and adjusted for various confounding factors, including medical care seeking, collection and testing of specimens, and reporting of cases. These methods are limited to medically attended illnesses and require relatively comprehensive data for accuracy, which leads to long periods of time between data collection and the availability of results (21). In this study, we directly surveyed for influenza and other respiratory pathogens in the community, allowing for a rapid assessment of pathogen characteristics and the associated clinical presentations among both care-seeking and non-care-seeking study populations. When combined with estimator models, on-the-ground surveillance of community-dwelling individuals with less severe illness and a wider range of demographic backgrounds may enhance our understanding of the burden of various respiratory pathogens in a community.

Similarly, estimator models with complete reliance on laboratory-confirmed cases can be limiting, especially during epidemics or pandemics in heavily affected regions where outbreak dynamics are rapidly evolving and the capacity of the health care system to adequately test cases has been exceeded (22). The benefits of direct, home-based surveillance among community-dwelling individuals can be seen in the context of the current COVID-19 pandemic. From 1 January 2020 to 9 March 2020, the Seattle Flu Study detected 78 cases of SARS-CoV-2 through direct sampling of community members, including the first documented case of community transmission in the United States, with 36 cases identified through the Swab and Send study (22, 23). This study enrolled and tested a large cohort of individuals with ARI symptoms across a large geographical area, of which half did not seek clinical care prior to or during the study period. The at-home study design proved to be an effective means of studying individuals infected with influenza and other respiratory pathogens, of whom many may not have been captured by traditional clinic or hospital surveillance. This design demonstrates that when faced with an emerging infectious disease, home-based testing can identify cases among non-care-seeking individuals, providing essential information for pandemic identification, spread, and management.

Limitations of this study include the enrollment of a study population that was not representative of the greater Seattle, WA, area. King County demographic data from the 2010 census shows that 49.8% of residents were male and 21.4% were 17 years of age and under, whereas our study population included 27.3% males and 7.7% minors. Additionally, the King County population is 6.0% black or African American and 8.9% Hispanic, whereas our study cohort was only 0.8% black or African American and 4.2% Hispanic. The median King County household income in 2016 was $78,800 per year, whereas the largest proportion (26.6%) of participants had a household income of greater than $150,000 per year (https://www.kingcounty.gov/∼/media/depts/executive/performance-strategy-budget/regional-planning/Demographics/Dec-2018-Update/KC-Profile2018.ashx?la=en). We hypothesize that factors related to a lack of Internet access and unfamiliarity with online systems may have contributed to lack of representativeness among certain groups in our study population. The utilization of targeted recruitment strategies aimed at enrolling a larger proportion of participants who were underrepresented in this cohort, including males, children, minorities, and individuals of lower socioeconomic statuses, could be implemented to yield a more representative study population. To encourage greater participation across the population, a stronger focus may be placed on recruitment measures, such as engagement with community-based organizations, that target a variety of demographic groups within the community rather than relying on untargeted social media and Internet advertisements for future implementations of this methodology.

Additionally, while most participants returned their home swab kits with no packaging or sample tube use errors at a rate concordant with a previous study (24), improvements to instructions (e.g., inclusion of instructional videos) may decrease these error rates. Further limitations of this study include use of self-collected midnasal swabs, which are not the gold standard for respiratory pathogen detection. However, our group has previously demonstrated that self-collected midnasal swabs are highly concordant with health care worker-collected nasopharyngeal swabs for the detection of SARS-CoV-2 (25), with results comparable to those of previous studies on the detection of viral pathogens by patient-collected midnasal swabs (20, 26–28). In addition, the contemporary control analysis included in this study shows that *C_rt_* values for pathogen-positive samples collected by health care workers are comparable to those of self-collected samples, with *C_rt_* values for health care-collected swabs lower for some targets but higher for others than self-collected swabs. Finally, the requirement of Internet access and delivery addresses that are easily accessible by standard shipping couriers may limit the scalability of this method in low resource or rural settings.

Our method for home-based respiratory pathogen surveillance can be scaled up to span larger geographic regions. When scaling up home-based surveillance, it will be important to ensure that individuals can receive a home swab kit within days of symptom onset and that nasal specimens can be returned to the laboratory in a timely manner. Depending on the geographic reach of the surveillance system, scaling up the study may require utilizing multiple fulfillment centers and laboratories, making logistics more complex. Quality-control measures to ensure consistency of test results across laboratories will then also be necessary. Another barrier to scale up this method lies in the challenges of obtaining the supplies needed to test more samples, as the availability of such supplies may be limited during pandemics.

Home surveillance of SARS-CoV-2 can be utilized to assist with the COVID-19 pandemic by scaling up the study methodology presented in this paper, collaborating with local public health departments, translating home swab kit instructions and online surveys into multiple languages, and obtaining Clinical Laboratory Improvement Amendments of 1988 (CLIA) certification, which is required to return COVID-19 test results. The use of home surveillance provides individuals with additional options for COVID-19 testing while reducing the risks associated with gathering at in-person testing centers. The Seattle Flu Study research group utilized these methods to assist with the COVID-19 pandemic by launching the Greater Seattle Coronavirus Assessment Network (SCAN) in partnership with Public Health—Seattle & King County in March 2020 (https://scanpublichealth.org).

In conclusion, at-home surveillance with self-collected nasal swabs is a feasible method to study the community-based prevalence of influenza during seasonal epidemics on a city-wide scale. This methodology can be adapted to study a variety of respiratory pathogens affecting diverse study populations with the ability to scale up to larger sample sizes. In particular, this approach allows for the inclusion of non-care-seeking individuals in respiratory pathogen surveillance studies and may be especially useful during epidemics or pandemics when quarantine and social distancing measures are in place to reduce transmission risks.

## Supplementary Material

Supplemental file 1
