# Supplementary material for: Evaluating Specimen Quality and Results from a Community-Wide, Home-Based Respiratory Surveillance Study
Source: J Clin Microbiol. 2021 Apr 20;59(5):e02934-20. doi: 10.1128/JCM.02934-20 (PMC8091861; doi:10.1128/JCM.02934-20)

Table A1. List of symptoms that were used in online questionnaires to screen individuals for eligibility. Selecting either acute cough *or* two or more concurrent *qualifying* symptoms were considered an acute illness episode and made an individual eligible for enrollment in the Swab and Send study.

Feeling feverish or warm *	Runny/stuffy nose or sneezing *
Headache *	Feeling more tired than usual *
New or worsening cough **	Muscle or body aches *
Chills or shivering [X]	Increased trouble with breathing *
Sweats [X]	Diarrhea [+]
Sore throat or itchy/scratchy throat *	Ear pain/ear discharge [+]
Nausea or vomiting *	Rash [+]

* A qualifying symptom for study eligibility for individuals of any age

** A qualifying symptom that is sufficient on its own for study eligibility for individuals of any age

[X] Not a qualifying symptom for study eligibility

[+] A qualifying symptom for study eligibility for individuals <18 years of age

Table A2. Pathogens for which all Seattle Flu Study respiratory specimens are tested using a TaqMan RT-PCR.

**Viruses**	**Bacteria**
SARS-CoV-2[1]	*Streptococcus pneumoniae*
Influenza A - H3N2	*Mycoplasma pneumoniae*
Influenza A - H1N1	*Chlamydia pneumoniae*
Influenza A - Pan	
Influenza B	
Influenza C	
Respiratory syncytial viruses A and B	
Parainfluenza viruses 1-4	
Coronaviruses 229E, NL63, OC43, and HKU1	
Adenovirus	
Rhinovirus	
Human metapneumovirus	
Human parechovirus	
Enterovirus[2]	
Enterovirus D68	
Human Bocavirus	

[1] SARS-CoV-2 was tested for using a stand-alone assay whereas the remaining pathogens were tested for using the Open Array assay

[2] All enterovirus species A, B, C, D, and G, including: all Coxsackie serotypes under species A, B, C; all Echovirus serotypes; and all Poliovirus serotypes (1-3).

Table A3: Virological characteristics of enrolled participants, October 16, 2019-March 9, 2020

	**Total (%)**
**Any positive test result (N=3,509)**	1232 (33.9%)
**Test result* (N=3,638)**	
Influenza A	392 (10.8%)
Influenza B	252 (6.9%)
Influenza C	2 (0.1%)
RSV	106 (2.9%)
SARS-CoV-2 (N=2,843)**	36 (1.0%)
hRV	379 (10.4%)
PIV (I-IV)	50 (1.4%)
hCoV	312 (8.6%)
hBoV	13 (0.4%)
AdV	36 (1.0%)
hMPV	77 (2.1%)
Enterovirus	24 (0.7%)
**Coinfection (N=3,638)***	74 (2.0%)
**RNase P C_{RT} value (N=3,629), Mean (SD)**	19.0 (3.4)

* Results are not mutually exclusive

** Note: Only samples collected on or after January 1, 2020 were tested for SARS-CoV-2.

Table A4: Contemporary control comparison of healthcare worker-collected to self-collected nasal specimens from October 2019 to March 2020

**Seattle Children's Hospital**	**RNase P (N=4,463)**	**Influenza (N=2,660)**	**RSV (N=856)**	**hCoV (N=580)**	**hRV (N=2,366)**
Mean (SD)	13.4 (2.6)	15.0 (6.0)	17.7 (8.0)	20.2 (6.8)	24.7 (7.2)
Median [Min, Max]	13.3 [5.6, 30.8]	13.8 [1.8, 24.9]	16.5 [1.8, 38.2]	19.7 [3.4, 39.8]	25.6 [2.3, 39.8]
**Swab and Send**	**RNase P (N=3,629)**	**Influenza (N=644)**	**RSV (N=106)**	**hCoV (N=312)**	**hRV (N=379)**
Mean (SD)	19.0 (3.4)	18.7 (4.9)	18.4 (5.1)	18.1 (5.1)	20.9 (4.6)
Median [Min, Max]	18.7 [9.6, 33.8]	18.9 [5.2, 27.7]	18.5 [17.8, 39.2]	17.8 [6.8, 27.7]	21.0 [8.14, 27.8]

Figure A1: *Quick Start Instruction Guide*

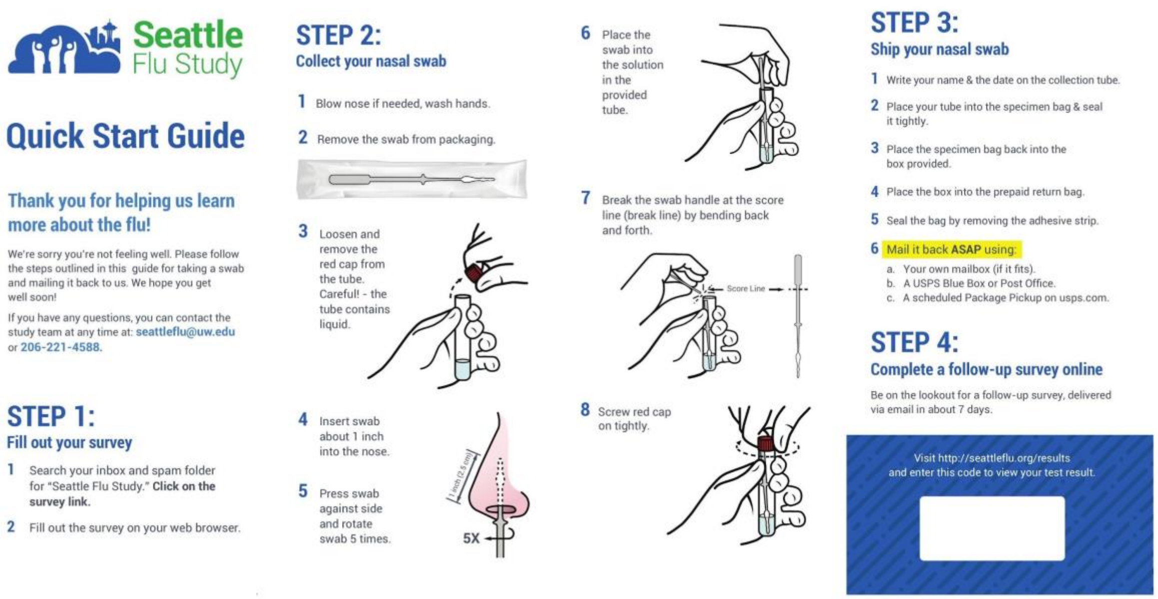

Supplement: Supplemental file 1 [file JCM.02934-20-s0001.pdf]
